# Gene target discovery with network analysis in *Toxoplasma gondii*

**DOI:** 10.1038/s41598-018-36671-y

**Published:** 2019-01-24

**Authors:** Andres M. Alonso, Maria M. Corvi, Luis Diambra

**Affiliations:** 1 Instituto de Investigaciones Biotecnológicas “Dr. Raul Alfonsin”, CONICET-Universidad Nacional de General San Martín, Chascomús, B7130IWA Argentina; 2 CREG, CONICET-Universidad Nacional de La Plata, La Plata, CP 1900 Argentina

## Abstract

Infectious diseases are of great relevance for global health, but needed drugs and vaccines have not been developed yet or are not effective in many cases. In fact, traditional scientific approaches with intense focus on individual genes or proteins have not been successful in providing new treatments. Hence, innovations in technology and computational methods provide new tools to further understand complex biological systems such as pathogen biology. In this paper, we apply a gene regulatory network approach to analyze transcriptomic data of the parasite *Toxoplasma gondii*. By means of an optimization procedure, the phenotypic transitions between the stages associated with the life cycle of *T*. *gondii* were embedded into the dynamics of a gene regulatory network. Thus, through this methodology we were able to reconstruct a gene regulatory network able to emulate the life cycle of the pathogen. The community network analysis has revealed that nodes of the network can be organized in seven communities which allow us to assign putative functions to 338 previously uncharacterized genes, 25 of which are predicted as new pathogenic factors. Furthermore, we identified a small gene circuit that drives a series of phenotypic transitions that characterize the life cycle of this pathogen. These new findings can contribute to the understanding of parasite pathogenesis.

## Introduction

**Figure 1:**
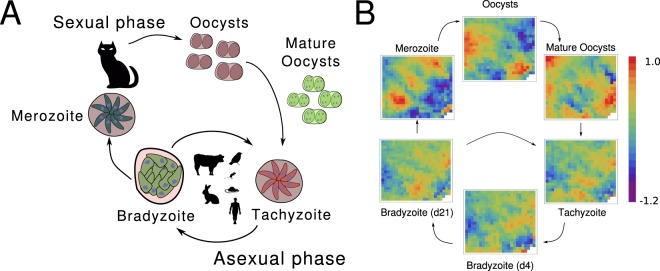
*Toxoplasma gondii* life cycle. (**A**) A schematic representation of the parasite biological cycle; (**B**) Expression profile of the parasite at the different life cycle stages. After a redundancy reduction procedure, we have found that the microarray dataset can be reduced to 545 clusters of genes. These variables can be represented in a heat-map of 22 × 25 cells. The color of each cell in the heat-maps represents the activity level of a cluster. The activity level of each cluster is given by the average of the expression levels of genes belonging to the cluster. The clusters position in the heat-maps is the same for all states, to facilitate the comparison between them.

Toxoplasmosis is a zoonotic disease that affects almost one third of the global population^1^. This condition is caused by the obligate intracellular parasite *Toxoplasma gondii*, which transits a complex life cycle. It develops an asexual phase in mammals and birds where the parasite cell can adopt an invasive and rapidly dividing form, the tachyzoite, and a latent form which is encysted in the host, the bradyzoite^2^. When bradyzoites are ingested by members of the *felidae* family, the definitive host, they differentiate into merozoite -an invasive and asexual form that will originate sexual gametocytes- and finally a sporulated form in oocysts, the sporozoite, as illustrated in Fig. 1A. Thus, the passage through the different life cycle stages allows the pathogen to adapt to diverse contexts by modulating its virulence and pathogenic potential^3^. While the stages of the biological cycle of *T*. *gondii* are characterized, the mechanisms that regulate the transitions between them are not completely understood. Different studies were directed to understand the phenomenon postulating that epigenetic regulation, changes in gene expression and subsequent activation/deactivation of genetic networks play a relevant role in the conversion from one stage to another^4^.

In order to understand how the *Toxoplasma* cycle is orchestrated, several systematic approaches have been implemented which are based on the application of high-throughput technologies (HTTs) in the field of epigenetics, genomics and proteomics. The protocols used include Chromatin immunoprecipitation (ChIP) in conjunction with microarray technologies (ChIP-chip)^5,6^, high-throughput sequencing (ChIP-seq) and gene expression studies based on microarray or sequencing technologies (RNA-seq)^7,8^. Given the range of experimental conditions and the typical performance of these techniques, a new challenge arises: organize and analyze resulting information from new technologies in a coherent framework. The methodologies mentioned above can provide almost complete observations of complex biological systems and can lead to a deeper understanding of the problem at the systems level. Consequently, understanding biological systems requires HTTs data products integration which are used to build quantitative models for *T*. *gondii*. Systems biology is an emergent and multidisciplinary field that proposes new and rational approaches for the analysis of HTT-derived information in the field of infectious diseases^9^. One of these goals involves the inference of gene regulatory networks (GRNs) from large amounts of information, since it allows modeling the dynamics of complex systems in a single conceptual framework^10–12^. GRNs are dynamic systems whose states are determined by the expression levels of each gene or groups of genes (nodes), while the edges, or links, between nodes represent regulatory interactions; the network architecture can be understood as a graph^13^. Once the network is reconstructed it is possible to address a number of different biological and biomedical questions such as the dissection of a key gene circuit involved in cellular differentiation^14^, the study of phenotypes related to health conditions, the development of new therapies, the design of perturbation experiments^15^ and interpretation of direct gene interactions such as transcription gene regulation through epigenomic data integration^16,17^. However, uncovering the GRN architecture represents a very difficult task, due to the limited amount of data available, many times affected noise, in comparison with the number of nodes in the network. Certainly, the fact that gene regulation involves feedback mechanisms and other nonlinearities, makes this challenge even more difficult^18^. In this sense, a computational techniques that allows for the reconstruction of a GRN from gene expression levels that overcome several major obstacles has been recently developed^12^ and applied to *T*. *cruzi*. Here we apply a GRN approach to study the *Toxoplasma gondii* life cycle, by integrating transcript expression data from sexual and asexual phenotypes as illustrated in Fig. 1B, obtained from the studies of Behnke *et al*.^19^ and Fritz *et al*.^20^, respectively. Despite the wide range of experimental conditions studied with different HTT, the only one that provides data on the life cycle of the parasite in a more comprehensive manner is still the microarray technology. This limitation could be overcome in the near future, allowing the integration of complementary data (for example, epigenetics) in more complete studies of this type.

Our proposed framework helps to reconstruct the network architecture which supports the six stages and the series of phenotypic transitions that make up the life cycle of *T*. *gondii*. The method was efficient to elucidate master key regulators involved in the analyzed phenotypical transitions. Most of the genes that are part of the subnetwork have not been characterized yet, while the presence of four dense granule proteins (GRA1, GRA2, GRA6, and GRA12) is highlighted. Finally, *in silico* perturbation experiments propose these key genes for future experimental studies in the tachyzoite to bradyzoite differentiation. Furthermore, by combining clustering methods and communities analysis it is possible to infer biological processes associated to these uncharacterized genes. While genes that are co-expressed tend to take part in the same processes and perform similar or complementary functions^18^, the inference of communities in the network allows to predict putative functions within the network.

We believe that the study of pathogen’s life cycles by gene network models leads to a thorough understanding of signaling pathways and their actors, being a powerful predictive tool for new molecular targets and diagnosis development as well as to assign functions to uncharacterized genes.

## Results

### Modeling the gene regulatory network of *T*. *gondii*

In order to model the *T*. *gondii* GRN we assume that the state of the system at time *t* can be represented by a *N*-dimensional vector *x*(*t*) associated with the expression levels of *N* clusters of genes, or nodes of the network. The dynamics of the network corresponds to a Markov model of order one, where the present state depends on the previous state in a linear fashion, following this equation:

1 $${x}_{i}(t+{\rm{\Delta }}t)=\sum _{j}\,{w}_{i,j}{x}_{j}(t)+{\theta }_{i}+{k}_{i}^{\mu }+{\varepsilon }_{i}(t).$$

Thus, the evolution of the system is governed by the matrix **W** and the external perturbations by **k**^*μ*^. The matrix elements *w*_*i*,*j*_ tell us about the strength and type of the influence of cluster *j* on cluster *i* (*w*_*ij*_ < 0 indicates inhibition, *w*_*ij*_ = 0 indicates absence of influence, while *w*_*ij*_ > 0 indicates activation). The influence of environmental cues on genes are represented by ${k}_{i}^{\mu }$.

The next step consists of determining which, and how, nodes are affected by the environmental cues. To this purpose, further to consider the available expression data, we also take into account known biological facts: (i) the life cycle of parasite has seven stages, but we include in our analysis transcriptional data available for five stages: immature oocysts, mature oocysts, tachyzoite, bradyzoite and merozoite; (ii) there are four possible transitions between these five stages, promoted by different environmental cues; (iii) The system fluctuates around one of attraction basins associated with the parasite stage; and (iv) the connectivity matrix is a sparse matrix. The available transcriptome data consist in six data points over the life cycle of *T*. *gondii*: oocysts day 0 (Od0), oocysts day 10 (Od10), tachyzoite day 2 (Tzd2), bradyzoite day 4 (Bzd4), bradyzoite day 21 (Bzd21), and merozoite of cat #52 (Mc52). Notice that the four external differentiation signals considered here, **k**^*μ*^ with *μ* = 1, 2, 3, 4, are associated to the following phenotypic transitions: Od0 → Od10, Od10 → Tzd2, Tzd2 → Bzd21 and Bzd21 → Mc52, respectively. The data point Bzd4 is part of the transition trajectory Tzd2 → Bzd21. We do not consider the transition Mc52 → Od0, since transcriptional data for micro- and macrogamete states are not currently available.

As described by Carrea *et al*.^12^, we perform the network reconstruction procedure in two steps. First, we focus on embedding the six data points into the dynamics of the network as steady states and on getting a connectivity matrix consistent with that. Then, considering the transitions between the different life cycle stages of the parasite and the previous connectivity matrix, we devise how external signals drive the phenotypic transitions.

### Learning about the steady states

**Figure 2:**
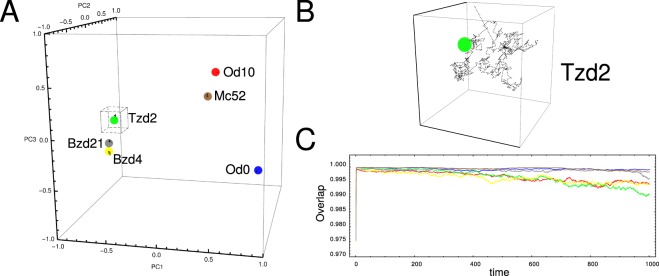
Embedding steady states. (**A**) The plot depicts the positions of the 6 stages of *T*. *gondii* represented by colored dots in the principal components space. First principal component accounts for 36.5% of the variance across the samples, while the second and third components explains 30.7% and 16.2%, respectively. Of course, the same variance percentages also correspond to the principal axes showed in Figs 5 and 7. The black lines around each stage correspond to the trajectories obtained using the model Eq. (1) without external signals. (**B**) Zoom view over the trajectory around the Tzd2 steady state. It can be observed that the state system fluctuates in the attraction basin associated with Tzd2 state; (**C**) Time course of the overlap between the system state at time *t* and the target stage: Od0 (blue), Od10 (red), Tzd2 (green), Bzd4 (yellow), Bzd21 (gray) and Mc52 (brown).

The first step is to embed the attraction basins, associated with each stage, into the dynamics of the GRN. For this purpose, we consider the temporal evolution of the network, described by Eq. (1), without the influence of environmental cues, and apply the singular value decomposition (SVD) method to compute connectivity matrix **W**, as indicated in the corresponding Methods section. Since the elements of the connectivity matrix vary continuously, most of the inferred matrix elements are close to zero. Consequently, the number of predicted edges of the GRN is quite high in comparison to the number of regulatory links in known biological networks^21,22^. With the aim to achieve a dilute version of **W**, i.e. a matrix in which most of the elements are zero, we considered a kind of bootstrap method. As such, we have added different realizations of noise to the states corresponding to each stages, and constructed 500 training sets. By computing the minimum *L*_2_ norm solution for each training set, we are able to calculate a histogram distribution, *P*(*w*_*i*,*j*_), for each element of the connectivity matrix. In this manner, we assessed the merit of the weighted values by performing a location test (*p*-values 0.01) as indicated in Methods section. Then, we clip the non-significant weights to construct a sparse version of the connectivity matrix, **W**_*ss*_, able to support the parasite’s stages as steady states. At this significance level, there are 17,410 edges between the 545 gene clusters, i.e., around 94% of the elements of **W**_*ss*_ are null. In order to quantify how the dynamics of the GRN with the matrix **W**_*ss*_, was able to capture the parasite’s stages as basins of attractions of the system, we calculated the overlap between the actual state and the target stage of the network. Figure 2A displays the trajectories illustrating the dynamics of the network around the steady stages. A zoomed view over the stage Tzd2 allows to appreciate that the course of the system (black lines) fluctuates in the basin of attraction associated with the Tzd2 stage indicated by the green dot, Fig. 2B. We perform simulations of the model for six different initial conditions chosen near to each stage and calculate the overlap between the state of the system at time *t*, and the states corresponding to the target stages for each simulation. Figure 2C displays the temporal behavior of these overlaps showing, in an alternative fashion, how the system can fluctuate around the basin of attraction associated to each parasite stage. This result suggests that once the system reaches an attraction basin it will move around within the basin as long as no external signal displaces the system from the basin. The size of fluctuations seems to vary from stage to stage leading to different perceptions of the size of basins. However, we observe that different initial conditions result in different size of fluctuations and to obtain a reliable estimation of size basins we need to run simulations for much longer periods and from many initial conditions. Getting a conclusion on the stability of the stage only inferred from the present analysis could not be reliable, since the dataset used here consists in only two biological replicates. Furthermore this analysis does not provide much information on the fluctuations of the system. Thus, we believe that this question could be properly addressed when single-cell RNAseq data for *Toxoplasma* becomes available.

**Figure 3:**
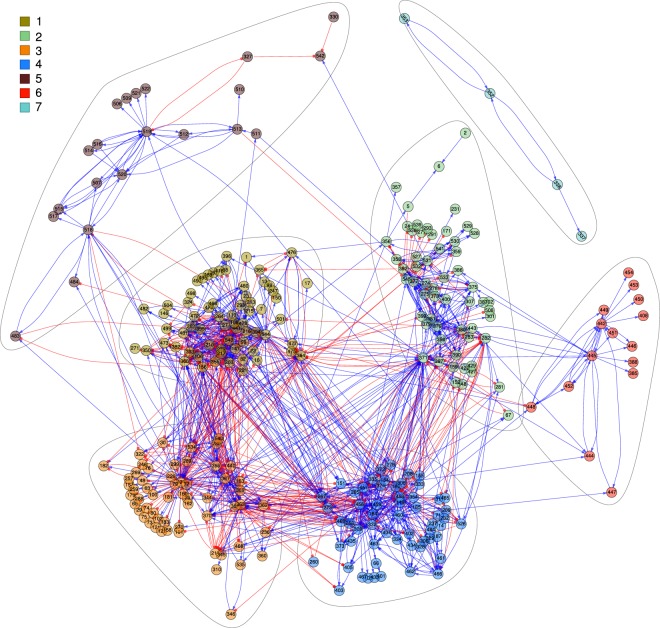
A directed graph representation of the *T*. *gondii* gene regulatory network. Nodes are grouped in seven communities. The color of the nodes identifies the membership with the corresponding community. By definition, nodes that are members of a community are more connected to each other than nodes belonging to other communities. Blue links represent up-regulation interactions between nodes, while red links represent down-regulation interactions. The arrows indicate the direction of regulation, i.e., from regulator to regulated. Additional details of communities and nodes are described in Fig. 4 and Supplementary Table S2.

The visualization of the obtained network is a challenging task, even with the low average node degree associated to our sparse GRN (~6%). In order to overcome this, we show only a selected fraction of nodes grouped in seven communities without considering the self-regulatory links. As result Fig. 3 displays 1358 links with small *p*-value (10^−130^), while the complete set of 17,410 links, including self-regulatory links, is listed in Supplementary Table S1. The communities analysis of the network, depicted in Fig. 3, grouped nodes by clumps of nodes that were more connected to each other than to the rest of the graph^23^; as a result nodes were grouped in seven communities with acceptable value of modularity (*Q* = 0.49). A more restricted threshold (*p*-value smaller than 10^−130^) reduces the average connectivity and increases the modularity of the community structure. Supplementary Fig. S1 depicts the modularity and the number of regulatory links in the resulting network when using different thresholds. Next, we analyzed gene ontology terms (associated molecular functions and biological processes) of the genes related to each community. In this manner, putative functions of uncharacterized genes can be inferred based on genes with a known function in the same community. A total of 338 of 708 genes in the network are uncharacterized, whereby processes and functions can be assigned based on the nodes with known functions that integrate the community.

**Figure 4:**
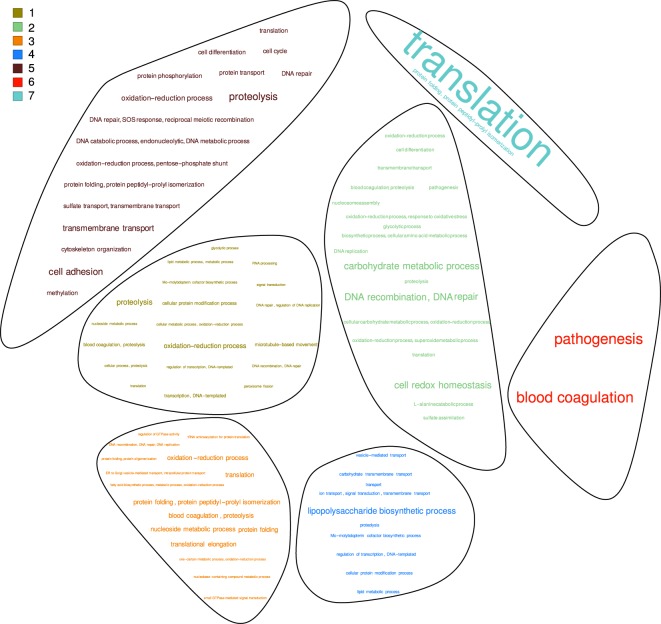
Main gene ontology terms of the communities. A word cloud plot resulting from the analysis of the gene ontology terms (biological processes) of the annotated genes belonging each community. Communities are composed by gene clusters (nodes) that participate in similar biological processes. The size of the words are proportional to the frequency of the term within community.

Nodes in community 1 participate, mainly, in oxidation-reduction and proteolytic process; 44 uncharacterized genes are part of this community. The analysis of community 2 indicates that 48 member genes with no known function, could participate in the DNA repair process. Community 3, that contains 66 uncharacterized genes, is integrated of genes associated to a variety of processes, but translational elongation stands out. Community 4 genes are mainly associated to the biosynthesis of lipopolysaccharides, and include 50 uncharacterized genes. Community 5 contains 105 unknown genes while the rest of the genes are associated with proteolysis and cell adhesion. Interestingly, genes from community 6 participate in pathogenesis, including 25 uncharacterized genes. Members of this last group should be studied in more detail since genes associated to this community might be novel pathogenic determinants. Finally, community 7 does not contain uncharacterized genes, but the gene products that integrate it participate in the translation process. By means of a word cloud representation we illustrate the results obtained from the graph analysis in an intuitive manner, Fig. 4 and Supplementary Table S2. In conclusion, combining clustering methods and graph structure analysis allows to systematically assign processes and functions to a large group of genes in the network.

Obtaining meaningful information from a network with more than 10,000 links can be a bottleneck in the genome-wide network analysis. One manner to overcame this difficulty is by considering only those nodes which are key for the maintenance of each stage of the life cycle. Inasmuch as regulatory function of a given gene relies on its activity level, there genes with a key regulatory role in a particular stage, but which are irrelevant in states when their activity level is almost null (i.e., *x*_*i*_~0). With this idea in mind, we have built for each steady state graphs which emphasize those nodes with a key role as regulators. In this sense we have displayed only those nodes that markedly regulate more than 5 other nodes. We have considered a regulatory interaction as marked when it explains more than 5% of the activity of regulated node, i.e., when |*w*_*i*,*j*_*x*_*j*_| ≥ 5% of |*x*_*i*_|. Thus, this feature depends not only on the weight of the link, but also on the current activity level of the regulator node. The Supplementary Figs S2–S6 depict the link-derived networks corresponding to parasite’s stages Od0, Od10, Tzd2, Bzd21 and Mc52, respectively. The main regulatory nodes represented in these plots are listed in Supplementary Table S3. We have found that the five analyzed stages share thirty-three of these nodes, twenty one of these nodes are present in the network showed Fig. 3, and are listed in Table 1.

**Table 1:** Regulatory clusters common to all the parasite’s stages.

Cluster ID	#genes	#marked links	Community	Molecular Function	Biological Process
1	1	67	1	microtubule motor activity (GO:0003777)	oxidation-reduction process (GO:0055114)
6	1	83	2	DNA repair (GO:0006281)	DNA recombination (GO:0006310)
75	14	2	3	nucleic acid binding (GO:0003676)	translational elongation (GO:0006414)
263	1	132	1	microtubule motor activity (GO:0003777)	oxidation-reduction process (GO:0055114)
327	4	66	5	aspartic-type endopeptidase activity (GO:0004190)	proteolysis (GO:0006508)
330	2	70	5	aspartic-type endopeptidase activity (GO:0004190)	proteolysis (GO:0006508)
359	1	66	2	DNA repair (GO:0006281)	DNA recombination (GO:0006310)
374	1	105	4	protein kinase activity (GO:0004672)	protein phosphorylation (GO:0006468)
442	5	102	6	protein kinase activity (GO:0004672)	pathogenesis (GO:0009405)
459	1	95	4	protein kinase activity (GO:0004672)	protein phosphorylation (GO:0006468)
474	2	178	3	nucleic acid binding (GO:0003676)	translational elongation (GO:0006414)
478	1	157	1	microtubule motor activity (GO:0003777)	oxidation-reduction process (GO:0055114)
501	1	52	1	microtubule motor activity (GO:0003777)	oxidation-reduction process (GO:0055114)
513	6	76	5	aspartic-type endopeptidase activity (GO:0004190)	proteolysis (GO:0006508)
518	1	97	5	aspartic-type endopeptidase activity (GO:0004190)	proteolysis (GO:0006508)
519	3	91	5	aspartic-type endopeptidase activity (GO:0004190)	proteolysis (GO:0006508)
520	1	73	5	aspartic-type endopeptidase activity (GO:0004190)	proteolysis (GO:0006508)
531	1	98	2	DNA repair (GO:0006281)	DNA recombination (GO:0006310)
540	1	67	3	nucleic acid binding (GO:0003676)	translational elongation (GO:0006414)
542	1	74	5	aspartic-type endopeptidase activity (GO:0004190)	proteolysis (GO:0006508)
545	1	134	2	DNA repair (GO:0006281)	DNA recombination (GO:0006310)

The communities to which each cluster belongs and the most representative gene ontologies are detailed.

### Modeling the phenotypic *T*. *gondii* transitions

**Figure 5:**
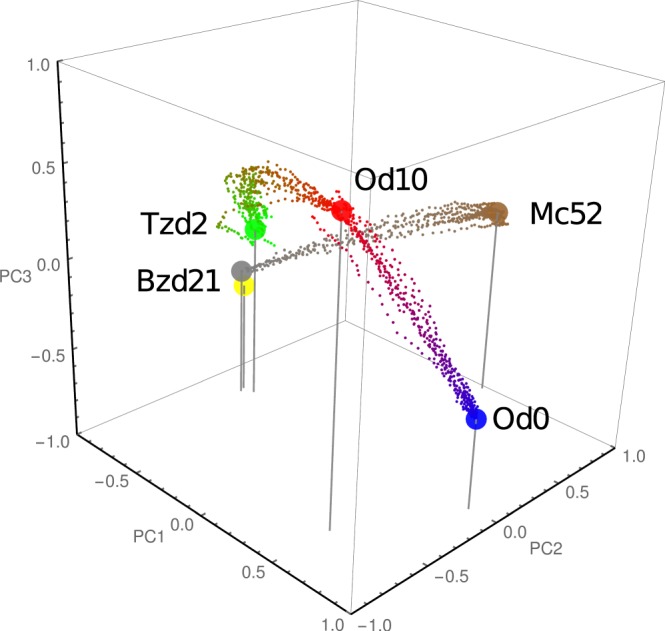
Environmental signals drive phenotypic transitions in *T*. *gondii*. The plot depicts three phenotypic transitions of the system under the influence of external cues obtained with our model Eq. (1), in the principal components space. Each transition comprises 44 time steps indicated by dots. In the plot we display 10 trajectories for each transition obtained with different noise realizations. Only the transitions Od0 → Od10, Od10 → Tzd2 and Bz21 → Mc52 are here represented. The transition Tzd2 → Bzd21 is better appreciated in Fig. 7A.

The second step in our analysis is to include in the GRN dynamics the phenotypic transitions between the stages embedded in the previous section. To this end we create a trajectories set, denoted by *D*_*t*_, that consider the shortest possible path that join the initial stage of the phenotypic transition and the associated ending state, as indicated in Methods section. For construction, the size of *D*_*t*_ is smaller than the size of the GRN (i.e., *M* < *N*), consequently there are boundless solutions consistent with *D*_*t*_. Among them we are interested in a particular one, the closest to the connectivity matrix **W**_*ss*_. Thus, the selected connectivity matrix, denoted by **W**_*t*_, can be found by:

2 $${{\bf{W}}}_{t}={{\bf{W}}}_{{D}_{t}}+{{\bf{C}}}_{ss}\cdot {{\bf{V}}}^{T},$$

where ${{\bf{W}}}_{{D}_{t}}$ is the solution of minimum norm in *L*_2_ computed by SVD for the trajectories set *D*_*t*_. **C**_*ss*_ is matrix numerically obtained by optimizing the overdetermined problem posed in the Method section; Eq. (10). In this manner, the obtained matrix **W**_*t*_ is compatible with the trajectories set associated to the phenotypic transitions, but moreover supports the multistability associated with the different life cycle stages of the parasite. To check how this connectivity matrix is able to reproduce the dynamics of the parasite during its life cycle we implement the model Eq. (1) with the connectivity matrix **W**_*t*_ to make simulations by considering different environmental cues *μ*. In each case, the network model simulations run by 44 times steps starting from one stage of the life cycle, and storing the states of the system at each time step. The time course of the 545 variables corresponding to the activity level of nodes (gene clusters) of the network can be illustrated by mean of the principal components or is compiled in a movie. In Fig. 5 we plot 10 alternative trajectories for the phenotypic transitions Od0 → Od10, Od10 → Tzd2, and Bz21 → Mc52, in the principal components space. Each trajectory, associated to a given simulated phenotypic transition is affected by identical environmental cue and start at the same initial state, however have a particular noise realizations. After 44 time steps the state of the system reached is consistent with the expected state considering the acting environmental cue. Alternatively, the complete temporal course of the system, from the oocyst to merozoite stage is compiled in a movie, which is available as Movie S1. Hence, the model can emulate the observed dynamical behavior of *T*. *gondii* during its life cycle.

In our model the external cues that drive the phenotypic transitions are represented by parameters **k**^*μ*^. To get insights on which genes could be modulated by environmental signals we have identified gene clusters associated with ${k}_{i}^{\mu }$-values greater than 95th percentile as those which are strongly activated by the acting environmental signals *μ*. In a similar manner, we identified those clusters associated with ${k}_{i}^{\mu }$-values lower than 5th percentile as the ones which are strongly inhibited by the same external cue. In this analysis we identify 140 gene clusters as externally regulated nodes, listed in Supplementary Table S4. This set comprises a total of 220 genes, 96 of which are still functionally uncharacterized. Interestingly, 40 of these genes are related with antigens, like microneme (MIC) proteins, dense granule (GRA) proteins, SAG-related sequence (SRS) proteins, and rhoptry proteins (ROP). Likewise, two genes that encode glycolytic enzyme enolases are indicated as externally regulated during transition Tzd2 → Bz21 by our analysis (clusters 364 and 376). It is important highlight that enolases are recognized as moonlight proteins, i.e., proteins that have dual functions^24,25^; in *T*. *gondii* enolases fulfill a second function as transcriptional regulators implicated in parasite differentiation and cyst formation^26,27^.

**Figure 6:**
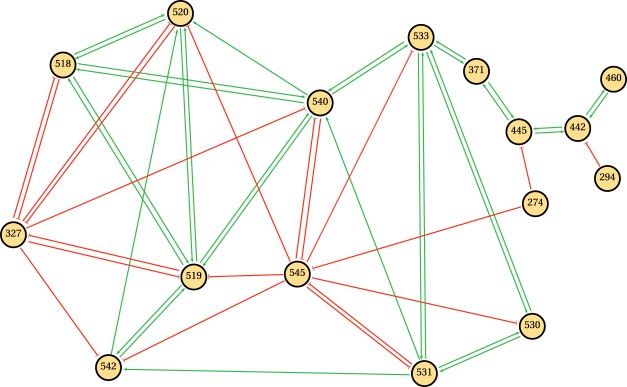
Subnetwork module associated with life cycle of *T*. *gondii*. This module is the minimal subnetwork that explains the studied phenotypic transition of *T*. *gondii*. Green links represent up-regulation between nodes, while red links represent down-regulation. The arrows indicate the direction of regulation, i.e., from regulator to regulated. Detailed information about node composition of this module can be found in Supplementary Tables S5 and S6.

Further analysis is conducted to identify the circuit that drives the state of the system along the life cycle of the parasite as result of the external cues. This step requires to identify an small set of regulatory links and cluster genes from a network with more than 17,000 links. The number of putative gene circuits within a network with this dimension is quite large and the assessment of all subnetworks can result in an unfeasible task. As such, we scale down the space of the search by considering only those subnetworks formed mainly by gene clusters with many links. To that purpose, we search for cyclic graphs, that contain only regulatory clusters, in matrix **W**_*t*_ and evaluate the ability of the module to emulate the dynamics associated with the parasite life cycle. In order to do that, we have reduce our model to a binary version of Eq. (1), where the variables *x*_*i*_ are binary and the system evolves following the equation:

3 $${x}_{i}(t+{\rm{\Delta }}t)=Sign(\sum _{j}^{\ast }{w}_{i,j}{x}_{j}(t)+{{\rm{\Theta }}}_{i}+{k}_{i}^{\mu }),$$

where$\ast$ indicates that summation runs over the nodes belonging to the module. As final result we are able to recover a subnetwork with sixteen nodes whose topology is illustrated in Fig. 6. The parameter values associated to this subnetwork are the same that the ones determined by Eq. (2), and they are listed in Supplementary Table S5. The subnetwork illustrated in Fig. 6 is able to reproduce many features of the dynamics of *T*. *gondii* life cycle, such as the phenotypic transitions Od0 → Od10, Od10 → Tzd2, Tzd2 → Bzd21 and Bzd21 → Mc52. The list of nodes that composes this subnetwork includes: 274, 294, 327, 371, 442, 445, 460, 518 519, 520, 530, 531, 533, 540, 542 and 545, which in turn comprises a total of twenty six genes. Fifteen of these genes still have no assigned known function, while the rest of genes already characterized include four genes associated with GRA proteins and other three genes associated with ribosomal proteins, and one coding for a redoxin domain-containing protein. Additional information about these clusters is given in Supplementary Table S6.

**Figure 7:**
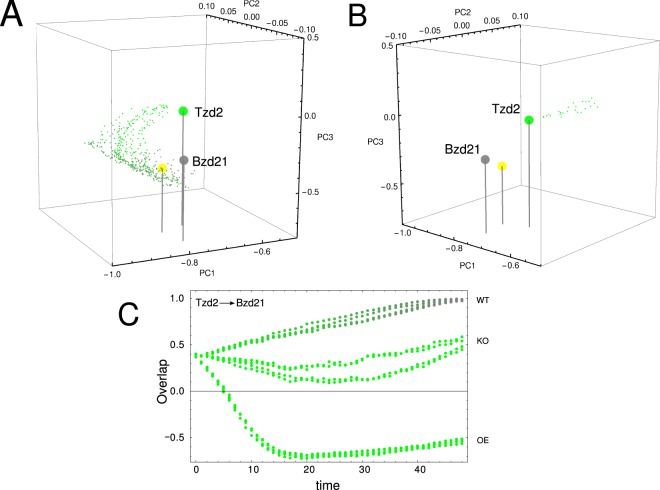
The role of node 274 on phenotypic transition Tzd2 → Bzd21. (**A**) A three principal components representation of Tzd2 → Bzd21 transition obtained with our model Eq. (1). Colored large dots represent the Tzd2 (green), Bzd4 (yellow) and Bzd21 (gray) states, while small dots represent transient states during the transition; (**B**) Trajectory of the system when node 274 was deleted. Our model predicts that with this mutation the system can not complete the Tzd2 → Bzd21 transition. (**C**) Time course of the overlap between the current state of the perturbed network and Bzd21 stage obtained with our model. We explore four alternative simulations of two different perturbations over node 274: over-expression (OE) and knock-out (KO).

**Figure 8:**
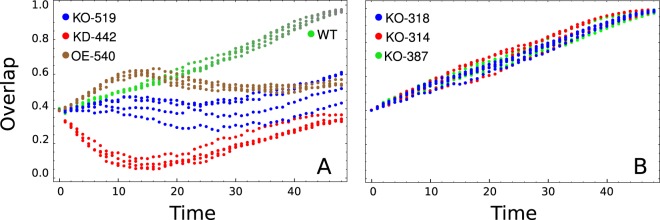
Perturbation experiments of transition Tzd2 → Bzd21. (**A**) Time course of the overlap between the network state at time *t* and the Bzd21 stage predicted by our model Eq. (1) for three perturbation over different nodes (519, 442 and 540) affecting the transition Tzd2 → Bzd21. (**B**) Time course of the overlap between the network state at time *t* and the Bzd21 stage predicted by our model for three different knock-outs: node 314 (red), node 318 (blue) and node 387 (green). Notice that these disturbances do not prevent the system from reaching stage Bzd21. In all cases dots represent transient states between the steady states.

Finally, we perform *in silico* perturbation experiments on the clusters of this module with the aim to confirm the relevant role of these nodes in the network dynamics. With the aim to identify relevant genes for the system’s dynamics and since bradyzoite phenotype has an important role for the development of the chronic disease^2^, the perturbation analysis is focused over the tachyzoite to bradyzoite transition. Table 2 summarizes the result of the complete perturbation experiment over all subnetwork nodes in this transition. Figure 7 illustrates the influence of perturbation of node 274 in such phenotypic transition. While Fig. 7A depicts the transition of wild-type (WT) in the 3-dimensional space of principal components, Fig. 7B illustrates that deletion or knock-out (KO), of the 274 node prevents the system from reaching the bradyzoite stage. Additionally, over-expression (OE) of this node drives the state of the system even far from the expected fate like is depicted in Fig. 7C; this is consistent with the expression matrix listed in Supplementary Table S7, line 276. Perturbations which are in the same direction of the WT does not impair the system to reach the expected fate. In this sense, Table 2 shows that there is always at least one perturbation without effect in the final fate, for example, the knock-down (KD) of the 274 node does not cause any effect because this node is down regulated during this transition as can be appreciated in Supplementary Table S7, line 276. Other examples are shown in Fig. 8A, where perturbation of nodes 519 (KO), 442 (KD) and 540 (OE) impairs the ability of the system to achieve the bradyzoite stage from tachyzoite. It should be noted that node 442 is composed mostly of genes that codify for dense granule antigens, see Supplementary Table S6. This observation is interesting since these antigens are proposed as important factors to the development of the asexual phase of the cycle, particularly in tachyzoite and bradyzoite stages^28^. In order to give significance to the above perturbation analysis of the module, we also perform perturbations on nodes which are not members of this subnetwork. In this sense we select at random 30 nodes with each one analyzed by perturbation in terms of knock-out, over-expression and knock-down during Tzd2 → Bzd21 transition. Figure 8B shows three examples of these control perturbations, where the system reaches the bradyzoite stage. We find that in only 6 cases (~6.6%) the perturbation is successful in impairing that system to reach the final fate. Thus, the perturbation experiments suggest that the nodes proposed here could be suitable candidates for master key regulators.

**Table 2:** Summary of *in silico* perturbation experiments over subnetwork nodes.

Cluster ID	KO	OE	KD
274	−	−	+
294	+	−	+
327	+	+	−
371	+	+	−
442	+	+	−
445	+	+	−
460	+	−	−
518	+	−	+
519	−	−	+
520	+	−	+
530	+	−	+
531	+	−	+
533	+	−	+
540	+	−	+
542	−	−	+
545	+	+	−

The experiment was performed on the transition from tachyzoite to bradyzoite steady states. The cases in which the system reaches the bradyzoite stage are indicated with the symbol “+” while those in which it is not reached are indicated with symbol “−”. KO: knock-out; OE: over-expression; KD: knock-down.

## Discussion

In this work, we integrated microarray expression data from five different phenotypes of the life cycle of *T*. *gondii* in a GRN model. The information of the phenotypic transitions between the different stages was used to implement a reverse engineering procedure which allowed us to reconstruct the connectivity matrix and determine parameter values linked to external modulations. From this matrix we identified a key network module that drives the phenotypical transitions, as well as the gene targets of the external modulations. In this way we embedded the dynamics of the pathogen’s life cycle in a high-dimensional network system. Analysis of the reconstructed network can help in the search of master regulators in the adaptation of *T*. *gondii* to different environments. This adaptation is the result of the expression of certain genes during each state of the cycle and can be explained by predicted regulatory relationships between gene clusters, providing us a blueprint that characterize each phenotype. In this sense, our work can contribute to the search of new antigens specific for each phenotype of *T*. *gondii* life cycle with potential applications as diagnosis tools, for example, to differentiate between acute and chronic infections.

Clustering methods have been traditionally used to infer functions of uncharacterized genes^18^. Basically, genes with known function grouped with not yet characterized one would allow inferring the functionality of the latter genes. In particular, experimental data have confirmed that genes participating in similar processes are co-expressed during the *T*. *gondii* replication cycle, even preserving the same *cis*-regulatory elements^29^. However, since of the 7,798 genes represented on the *T*. *gondii* chip 3,671 are not characterized, many clusters were completely integrated by uncharacterized genes. This feature, common in non-model organisms, can impair the gene function prediction task, via clustering methods. Alternatively, in a previous study on the Apicomplexa *Plasmodium falciparum*, authors have assigned functions to thousands of uncharacterized gene modeling the parasite interactome, by using the Bayesian approach^30^. Here we performed a further analysis based on communities in the gene interaction network^23^ to improve the gene annotation task. The identification of communities in a graph and the subsequent study of the structure of these communities allow to determine functional motifs within a molecular network^31,32^. Our enrichment analysis over the biological process of every gene in each community reveals that particular processes are predominant in different communities. By combining clustering and communities analyses it could be possible to infer the biological processes of uncharacterized genes. Using this analyses over a net of 708 genes grouped by clusters, Fig. 3, we were able to predict the function of 338 uncharacterized genes. Thus, our extended gene clustering procedure could be useful to predict common *cis*-regulatory elements, design experiments for determination of protein-DNA interactions, and to improve our current knowledge of the transcriptional regulatory network, as previously reported^13,16^.

Furthermore, our framework was able to predict a module that governs transitions between *T*. *gondii* steady states. This key network module is formed by sixteen clusters that could explain transitions between steady states. Most of these genes that integrate the master regulator of *T*. *gondii* are uncharacterized proteins. We have highlighted in this module the presence of dense granule proteins, as components of the cluster 442. GRA proteins constitute a group of relatively small proteins that are important for the development and metabolism of the parasitophorous vacuole, a highly dynamic compartment defining the replication permissive niche for the actively growing tachyzoite form of the parasite^28,33,34^. Our *in silico* perturbations experiments confirm that knock-out or over-expression of the cluster 442 do not prevent transition from tachyzoite steady state to bradyzoite steady state but knock-down of these genes could affect parasite cell fate when tachyzoite to bradyzoite transition is evaluated in our model. This observation is consistent with previous published results for GRA6 protein, where a biological role in cyst differentiation is proposed^35^. In addition, previous studies on a mutant ΔGRA2 strain are interesting since it tends to develop cysts *in vivo* unlike the wild-type counterpart^36^; while GRA1 is an essential factor for host invasion and replication^37,38^. A similar perturbation analysis over thirty clusters selected at random, have scarce ability to alter the dynamics of the system, supporting the key regulatory role proposed by our model.

In conclusion, in this work we confirm that our previous mathematical approach can be extrapolated to other protozoan pathogens allowing to reveal a subnet of master regulators that explain the dynamics of the transitions between the different phenotypes of *T*. *gondii*. These findings suggest that genes coding for GRA proteins could have a key role as regulators in tachyzoite to bradyzoite differentiation. This result is in agreement with a former study and reinforces the postulated role of GRA proteins in bradyzoite cysts development^39^. Consequently, experimental data based on perturbation experiments of the modeled network are necessary to confirm these observations. Finally, the methodologies here employed for the analysis of the modeled GRN could be useful to predict processes and functions of uncharacterized genes.

## Methods

### Data normalization

In this work we have used two microarray experiments made with the same chip^40^ and performed over type II clonal strains, M4 and TgNmBr1. In one of the studies Fritz *et al*.^20^ presents transcriptomic series of *in vitro* tachyzoite, *in vivo* and *in vitro* bradyzoite and complete oocyst development. On the other hand, in the second study Behnke *et al*.^19^ describes global gene expression of merozoite stage and integrate his results with the data obtained in the first study. Data sets are comparable^19^ and are accessible on GEO-database (Accession no.: GSE32427 & GSE51780). These experimental series represent expression analysis of five of the seven stages that comprises the life cycle of *T*. *gondii*. Expression was evaluated per replicate at different times for each state and we selected the following data sets to analyze: oocysts day 0 (Od0) and oocysts day 10 (Od10, mature oocysts), tachyzoite day 2 (Tzd2), bradyzoite day 4 (Bzd4) and bradyzoite day 21 (Bzd21), and merozoite of cat #52 (Mc52). The chip used in both studies provide whole genome expression profiling, using at least 11 perfect match probes for each of the ~8000 genes in the *T*. *gondii* genome, including both the apicoplast and mitochondrial genomes. Its also includes a variety of controls (actin, hypoxanthine-xanthine-guanine phosphoribosyl transferase, yeast housekeeping genes and mismatch probes), immune effector molecules (cytokines, receptors, etc.), and genes whose expression is suspected from previous studies to be altered by infection. More information about microarray can be found in the web^41^ and in^40^. Microarray data of the two data sets were loaded into R software using the *affy* package from Bioconductor Project and processed using Robust Multi-array Average (RMA) and quantile normalization^42^. The relative signal recorded at stages *α* = 1, 2, 3, 4, 5, 6, for the probe *i* and biological replicates *j* = 1, 2, was denoted by ${y}_{i}^{{\alpha }_{j}}$. These relative intensities were averaged over all replicates, i.e, ${\bar{y}}_{i}^{{\alpha }_{j}}=\frac{1}{2}{\sum }_{j}\,{y}_{i}^{{\alpha }_{j}}$. Control data was eliminated and only expression data from specific *T*. *gondii* probes were analyzed, which give us a normalized expression set of 7,798 probes for each sample. Thus, the expression level at time point *α* for the probe *i* is the quantity denoted by ${x}_{i}^{\alpha }=\,\mathrm{ln}\,[{\bar{y}}_{i}^{\alpha }/{\langle {\bar{y}}_{i}^{\alpha }\rangle }_{\alpha }]$. Supplementary Table S8 provides the normalized expression levels, ${x}_{i}^{\alpha }$, for each probe *i* and stage *α*, used in the next step.

### Redundancy reduction procedure

With the aim of reducing the redundancy in the experimental data set, we use an agglomerative hierarchical clustering method to group genes with similar expression levels. In particular, we use an unweighted pair group method known as UPGMA. The clustering procedure is halted when it reach a number of clusters, *N*_*c*_, that is convenient for the data-set under study, but which is not known in advance^43^. In order to estimate *N*_*c*_, we perform the agglomerative procedure for different *N*_*c*_ values, and calculate a measure of the clustering merit, known as Davies-Bouldin index (DBI)^44^. This index is defined as:

4 $$E=\frac{1}{{N}_{c}}\,\sum _{j=1}^{{N}_{c}}\mathop{\,{\rm{\max }}}\limits_{k\ne j}(\frac{{\delta }_{k}+{\delta }_{j}}{\parallel {c}_{k}-{c}_{j}\parallel }),$$

where $\parallel {c}_{k}-{c}_{j}\parallel$ denotes the distance between the centroids of clusters *k* and *j*, and ${\delta }_{k}={N}_{k}^{-1}{\sum }_{i}\parallel {c}_{k}-{x}_{i}\parallel$ is a measure of the gene dispersion within the cluster *k*, which has *N*_*k*_ genes. Low DBI-values indicate good cluster structures. However, we can always obtain lower DBI-values just by increasing *N*_*c*_ enough. Consequently, the adequate value of *N*_*c*_ must be a trade-off that involves a balance between accuracy and redundancy reduction. Supplementary Fig. S7 depicts the DBI *versus* the number of cluster for the data set used here. One can see that the clustering merit presents a local minimum at *N*_*c*_ = 545, and because of this we chose this value as the suitable *N*_*c*_ for the agglomerative procedure. As a result, the expression values of the 7,798 genes were organized in 545 clusters, and the intra-cluster averages (i.e., ${\bar{x}}_{j}^{\alpha }={\langle {x}_{i}^{\alpha }\rangle }_{i\in clusterj}$) were taken as dynamical variables for the subsequent modeling. The cluster membership of each gene is listed in Supplementary Table S9, while Supplementary Table S7 gives the mean values of the expression levels, ${\bar{x}}_{j}^{\alpha }$ for each stage *α* and cluster *j*. The resulting average levels, ${x}_{j}^{\alpha }$, corresponding to the six stages of life cycle of *T*. *gondii* are illustrated in 2D array plots of Fig. 1B.

### Reverse engineering methods

#### The gene network model and parameter estimation

In this study we use a linear model for the network, as in other previous works that have dealt with temporal profiles of expression data^18,45–47^. In particular, we implement this model in the framework of continuous variables but with discrete time for the evolution. This framework has two interesting advantages: (i) the assessment of model parameters does not have a high computational cost^18^, and (ii) it can take into account additive fluctuations. In this network model, the system state at time *t* is determined by the activity level of the *N* clusters of genes forming the network, denoted by the vector ${\bf{x}}(t)=({x}_{1},{x}_{2},\ldots ,{x}_{N})$. The equation governing the temporal evolution of the linear GRN can be written as:

5 $${x}_{i}(t+{\rm{\Delta }}t)=\sum _{j}\,{w}_{i,j}{x}_{j}(t)+{\theta }_{i}+{k}_{i}^{\mu }+{\varepsilon }_{i}(t),$$

where we have added a white Gaussian noise term, *ε*(*t*). *w*_*i*,*j*_ are the weights of the regulatory links present in the connectivity matrix **W**, *θ*_*i*_ is a constant that indicates how much the gene cluster *i* is expressed in the lack of inputs, and ${k}_{i}^{\mu }$ is the impact of the external signal cue *μ* over gene cluster *i*. Notice that we can write the predicted state of cluster *i* in a more compact manner:

6 $${x}_{i}(t+{\rm{\Delta }}t)=({w}_{i,1},{w}_{i,2},\ldots ,{w}_{i,N},{\theta }_{i},{k}_{i}^{\mu }).({x}_{1},{x}_{2},\ldots ,{x}_{N},1,1),$$

where *μ* corresponds to the acting external signal and parameters *θ*_*i*_ and ${k}_{i}^{\mu }$ have been used to extend the matrix **W**. We assume that our available gene expression data can be represented by *M* pairs of input-output, defining the training set *D* = {**X**, **Y**}. The columns of the input matrix **x**^*μ*^, which represents the state of the system at time *t*, can be mapped by the model to the columns of the output matrix, that is:

7 $${{\bf{y}}}_{v}={\bf{W}}{{\bf{x}}}_{v}\,v=1,\ldots ,M,$$

where **y**_*v*_ is the state of the system at time *t* + Δ*t*. To compute the matrix **W** that performs this mapping we minimize the cost function ${\sum }_{v}\parallel {y}_{v}^{\ast }-{y}_{v}\parallel$, where ${y}_{v}^{\ast }$ is the predicted state, ${y}_{v}^{\ast }={\bf{W}}{{\bf{x}}}_{v}$. This minimization problem has many alternative solutions when *M* < *N*, one of which is the minimum *L*_2_–norm solution, denoted here by ${{\bf{W}}}_{{L}_{2}}$. This solution can be written as ${{\bf{X}}}^{T}={\bf{U}}\cdot {\bf{S}}\cdot {{\bf{V}}}^{T}$, where **U**, **S**, and **V** are the matrices of the singular value decomposition of **X**^46,48^. Thus, the minimum *L*_2_–norm solution ${{\bf{W}}}_{{L}_{2}}$ is given by:

8 $${{\bf{W}}}_{{L}_{2}}={\bf{Y}}\cdot {\bf{U}}\cdot {\rm{diag}}({s}_{j}^{-1})\cdot {{\bf{V}}}^{T}.$$

Unfortunately, when we are dealing with GRN the number of genes is usually larger than the number of experiment (i.e., $M\ll N$) and there is an infinite number of solutions compatible with the training set *D*. However, there exist a closed-form expression for all solution of Eq. (7) in terms of ${{\bf{W}}}_{{L}_{2}}$:

9 $${\bf{W}}={{\bf{W}}}_{{L}_{2}}+C\cdot {{\bf{V}}}^{T},$$

where elements *c*_*ij*_ are 0 if *s*_*j*_ ≠ 0, otherwise they have arbitrary values. Following previous studies^12,46^ we can take advantage of this arbitrariness. First, we use the minimum *L*_2_–norm solution ${{\bf{W}}}_{{L}_{2}}$ to insert the six stages of *T*. *gondii* as steady states of the network dynamics. This will be described in the next subsection. Second, we take into account the knowledge about phenotypic transitions to reveal the influence of environmental signals. For this purpose we use Eq. ((9)) and the minimum *L*_2_–norm solution ${{\bf{W}}}_{{L}_{2}}$ computed in the first step.

#### Embedding the steady states

In this first step of the inferring procedure we construct a training set *D*_*ss*_ with size *M*. To this end, different noise realizations associated with each stage *α* were added to obtain the columns of input and output matrices as follows:

$$\begin{array}{c}{{\bf{x}}}^{v}=\{{\bar{x}}_{j}^{\alpha }\}+\{{\varepsilon }_{j}^{i}\},\\ {{\bf{y}}}^{v}=\{{\bar{x}}_{j}^{\alpha }\}+\{{\varepsilon }_{j}^{i^{\prime} }\}\,{\rm{with}}\,j=1,\ldots ,N,\end{array}$$

where the index *α* runs from 1 to 6, the index *v* runs form 1 to *M* and the indexes *i* and *i*′, which correspond to different realizations of noise, run from 1 to 50; consequently *M* = 6 × 50 = 300. *ε*_*j*_ is taken from a Gaussian distribution ($\bar{\varepsilon }=0$ and *σ*_*ε*_ equal to 1% of the standard deviation of the data). This procedure extends the size of the training set and allows the network to have a dynamic similar to that of the behavior of the parasite during its life cycle.

In order to construct a connectivity matrix with a low connectivity degree we need to discriminate whether the estimated matrix elements *w*_*i*,*j*_ are 0 or a value significantly different from 0. To this end, we have constructed a number of 500 training sets and computed the associated solution to each set. From this set of 500 slightly different solutions we have computed the histogram distribution for each weight, *P*(*w*_*i*,*j*_). Then, we implemented a location test for the distributions *P*(*w*_*i*,*j*_), as described in^12^. After that, we set to zero all weights with *p*-value greater than 0.01, otherwise the hypothesis is accepted, the assigned to *w*_*i*,*j*_ the average of the distribution. In this manner we construct a sparse matrix, denoted hereafter by **W**_*ss*_, which is consistent with the set of states present in *D*.

#### Embedding the phenotypic transitions

In the second step, we extend our analysis to insert the phenotypic transitions and determine the environmental cues that drive the transitions. We assume that transitions between states take place progressively passing through transient states along the shortest trajectory that link the initial stage *α* and final stage *β*. In this manner, when the system is driven by external cue, from state *x*^*α*^ to the target state *x*^*β*^, it makes successive transitions between transient states. The succession of these transient states, denoted by *x*^*α*,*β*^(*t*), can be constructed by:

$${{\bf{x}}}^{\alpha ,\beta }(t)=(({n}_{i}-t){{\bf{x}}}^{\alpha }+t{{\bf{x}}}^{\beta })/{n}_{i}\,{\rm{with}}\,t=0,1,2,\ldots ,{n}_{i}.$$

In order to embed the transitions $\alpha \to \beta$ into the network dynamics, we make a further training set, denoted by *D*_*t*_, by means of the transient states **x**^*α*,*β*^(*t*). The matrix’ columns **x**^*v*^ and **y**^*v*^ are given by:

$$\begin{array}{c}{{\bf{x}}}^{v}=\{{\bar{x}}^{\alpha ,\beta }(t)\}+\{{\varepsilon }_{j}^{i}\},\\ {{\bf{y}}}^{v}=\{{\bar{x}}_{j}^{\alpha ,\beta }(t+1)\}+\{{\varepsilon }_{j}^{i^{\prime} }\},\,{\rm{with}}\,t=0,1,2,\ldots ,{n}_{i}-1.\end{array}$$

In this paper we have considered four phenotypic transitions: Od0 → Od10, Od10 → Tzd2, Tzd2 → Bzd21, and Bzd21 → Mc52. The transition Tzd2 → Bzd21 includes the stage Bzd4 as part of the transition trajectory. For each transition, we consider 44 small transitions, i.e. *n*_*i*_ = 44, bringing the size of *D*_*t*_ to *M* = 176. Since size of *D*_*t*_ is smaller than the number of clusters *N*, there are many solutions consistent with this training set. Among all of them we are interested in the solution which is the nearest to the previously determined **W**_*ss*_. In order to determine this solution, we computed the smallest *L*_2_ norm solution associated to *D*_*t*_, denoted by ${{\bf{W}}}_{{D}_{t}}$, and by using Eq. (9) we estimate the matrix **C**_*ss*_ by mean of the equation:

10 $${{\bf{W}}}_{ss}={{\bf{W}}}_{{D}_{t}}+{{\bf{C}}}_{ss}\cdot {{\bf{V}}}^{T}.$$

Determining the elements of matrix **C**_*ss*_ from Eq. (10) is an overdetermined problem. We address this optimization problem by using the interior point method as in^12,46^. Then, the elements of matrix *C* so obtained were used to calculate the particular solution, represented by **W**_*t*_, by using ${{\bf{W}}}_{t}={{\bf{W}}}_{{D}_{t}}+{{\bf{C}}}_{ss}\cdot {{\bf{V}}}^{T}$. This new connectivity matrix is compatible with the phenotypic transitions present in training set *D*_*t*_, and it is also the most similar solution to **W**_*ss*_.

### Community analysis

One innovative concept for network analysis is known as community structures. Communities can be defined as groups of nodes with many edges joining nodes within the same group and comparatively few edges joining nodes of different groups or communities. To find communities on the regulatory network obtained in the previous inference process we use the method of random walk edge betweenness, proposed by Newman and Girvan^23^. This method is based on the concept of *edge betweenness*, which is defined as the number of shortest paths between node pairs that run along this edge, summed over all node pairs. Briefly, the Newman-Girvan algorithm involves calculating the betweenness of all edges in the network and removing the one with highest betweenness. By repeating this process the groups are separated from one another and the underlying community structure of the network is revealed. This analysis was implemented with the R-package *Community Detection using Modularity Suite*^49^. The procedure above leads to some partition of the network into communities even in networks without a significant community structure. For this reason, a measure of the goodness of the structure found is mandatory. For this purpose we used in this paper a measure called modularity^23^ which is defined as:

11 $$Q=\sum _{i}^{N}\,({e}_{ii}-{(\sum _{j}^{N}{e}_{ij})}^{2}),$$

where the element *e*_*ij*_ is the fraction of all links in the network that connect nodes in community *i* to nodes in community *j* and *N* is the number of communities in the network. The modularity ranges in values from 0, when number of intra-community edges is equal or less than a in random network, to 1 which corresponds to the strongest community structure.

## Electronic supplementary material

Supplementary Info

dataset 1

dataset 2

dataset 3

dataset 4

dataset 5

dataset 6

dataset 7

dataset 8

dataset 9

Supp movie 1
