# Supplementary material for: Gene target discovery with network analysis in Toxoplasma gondii
Source: Sci Rep. 2019 Jan 24;9:646. doi: 10.1038/s41598-018-36671-y (PMC6345969; doi:10.1038/s41598-018-36671-y)
Supplement: Supplementary file 1 — Supplementary Info [file 41598_2018_36671_MOESM1_ESM.pdf]

Gene target discovery with network analysis in *Toxoplasma gondii*

Andres M. Alonso[1,2], Maria M. Corvi[1], Luis Diambra[2]

[1] Instituto de Investigaciones Biotecnológicas "Dr. Raul Alfonsin", CONICET-Universidad Nacional de General San Martin, Chascomús B7130IWA, Argentina.

[2] CREG, CONICET-Universidad Nacional de La Plata, La Plata, CP 1900, Argentina.

Legends for Supplementary Tables and Movies

Supplementary Table S1: List of most significant regulatory links (p-value smaller than 0.01) needed for each stage of parasite to be a steady state. The table is organized as follows: IDs of the regulatory clusters (first column), IDs of the regulated clusters (second column), average value (third column) and standard deviations (fourth column) of the weight associated to the link, p-value of location test (fifth column).

Supplementary Table S2: List of genes grouped by related functions in 7 communities depicted in Fig. 3.

Supplementary Table S3: Information about key genes for the steady state maintenance (UniProt ID, ToxoDB ID, protein description, chromosome localization).

Supplementary Table S4: Information about genes most affected by environmental signals (UniProt ID, ToxoDB ID, protein description, chromosome localization).

Supplementary Table S5: Values of parameters $w_{i,j}$, Θ_i, and k_i^{μ} for the subnetwork module shown in Fig. 5.

Supplementary Table S6: Master key regulators. List of genes that integrate the clusters of the subnetwork that regulates the *T. gondii*'s life cycle.

Supplementary Table S7: Activity levels of gene clusters for each stage used in our model.

Supplementary Table S8: *T. gondii*'s gene expression profile used in the study. This table is organized as follows: probe IDs (first column), Uniprot IDs (second column), ToxoDB IDs (third column), protein description, as indicated in ToxoDB (fourth column). The remaining columns list the relative expression levels (log-normalized) of 7,798 probes included in the *T. gondii* microarray for 6 different stages: oocyst d0, oocyst d10, tachyzoite d2, bradyzoite d4, bradyzoite d21, merozoite.

Supplementary Table S9: Description of members of each cluster is organized as follows: gene IDs (first column), cluster IDs to which the gene belongs (second column). Probe IDs (third column), Uniprot IDs (fourth column), ToxoDB IDs (fifth column), protein description, as indicated in ToxoDB (sixth column).

Supplementary Movie S1: Animated representation of gene expression profile during parasite life cycle. The color of each cell represents the activity level of a gene cluster. The activity level of each cluster is given by the average expression levels of genes belonging to the cluster. In order to improve the visualization, the position of clusters in the array is assigned following a criteria of similarity of their activities. This order is fixed during the evolution. The animation is constructed by compiling 184 network states during the simulations of the system in the transitions: Od0 $\rightarrow$ Od10, Od10 $\rightarrow$ Tzd2, Tzd2 $\rightarrow$ Bzd21 and Bzd21 $\rightarrow$ Mc52.

Supplementary Figures

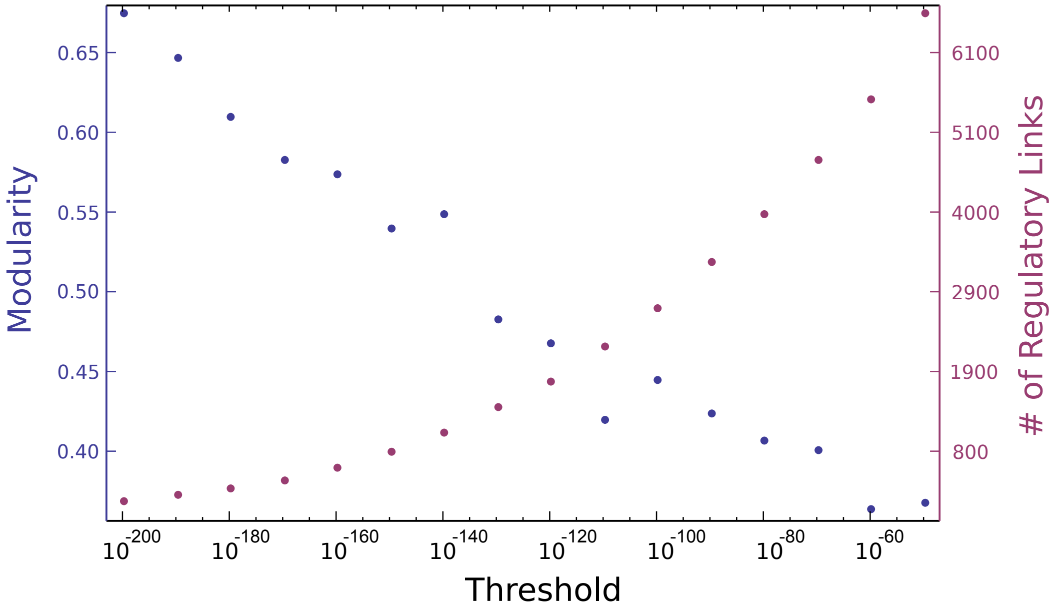

Supplementary Figure S1: Modularity (blue dots) and the number of regulatory links (red dots) in the network obtained by using different thresholds. With smaller threshold the network only includes links with the lower p-value, reducing the average connectivity and increasing the modularity of the community structure.

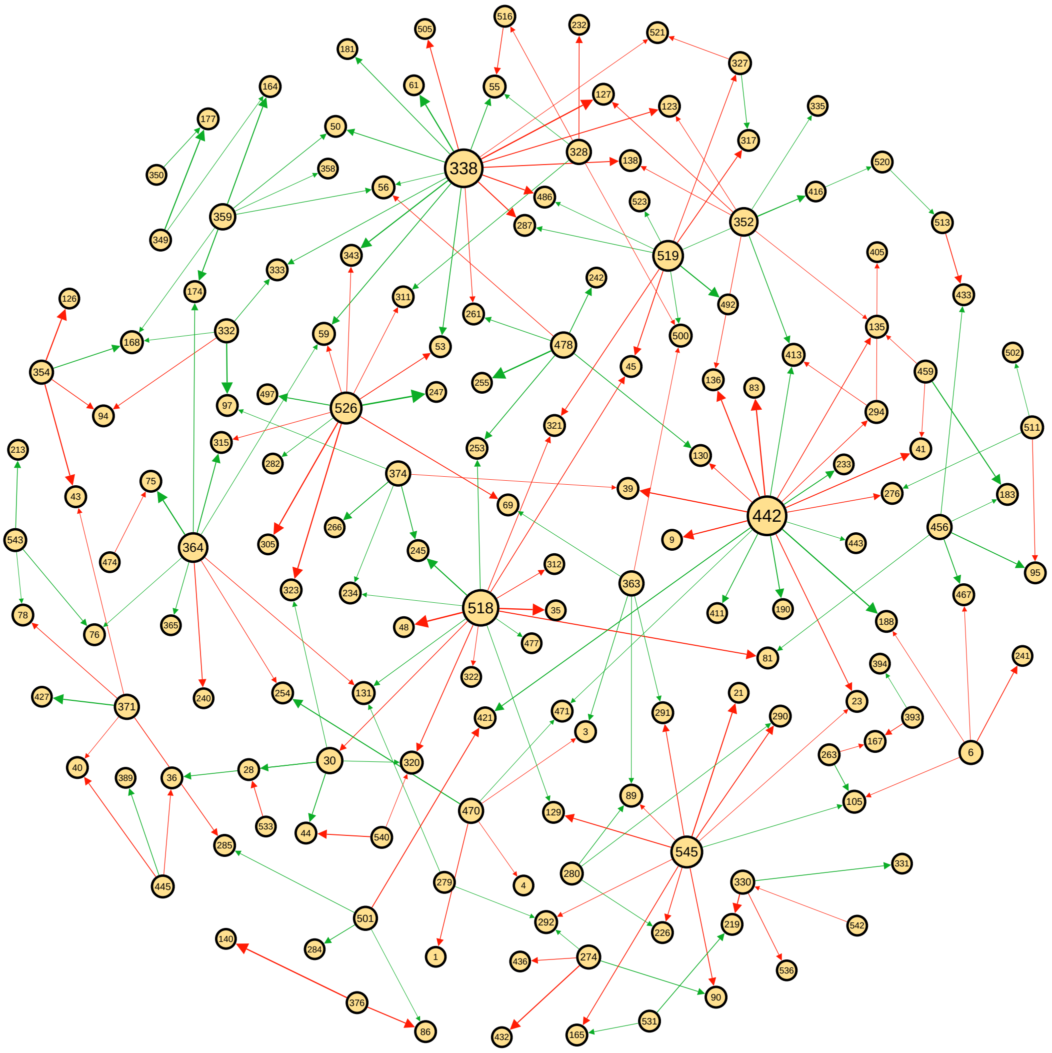

Supplementary Figure S2: Main regulatory clusters of Od0 state. Green links represent up-regulation interactions between nodes, while red links represent down-regulation interactions. The arrows indicate the direction of regulation, i.e., from regulator to regulated. The size of the cluster (circles) is proportional to the number of clusters that regulate. The size of the arrow is proportional to the fraction of the activity of regulated cluster explained by the influence of the regulator, i.e., proportional to $|w_{i,j}x_j|/|x_i|$.

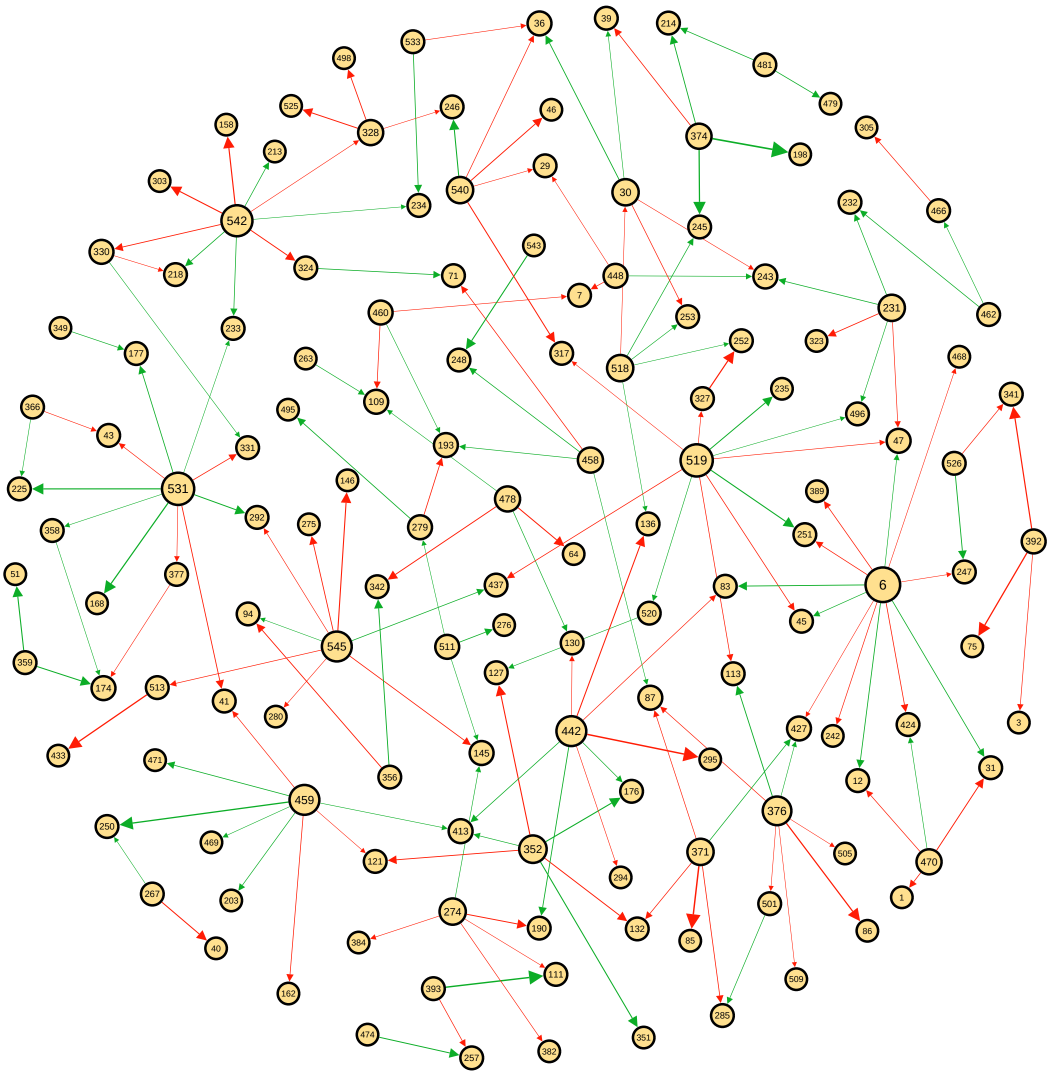

Supplementary Figure S3: Main regulatory clusters of Od10 state. Green links represent up-regulation interactions between nodes, while red links represent down-regulation interactions. The arrows indicate the direction of regulation, i.e., from regulator to regulated. The size of the cluster (circles) is proportional to the number of clusters that regulate. The size of the arrow is proportional to the fraction of the activity of regulated cluster explained by the influence of the regulator, i.e., proportional to $|w_{i,j}x_j|/|x_i|$.

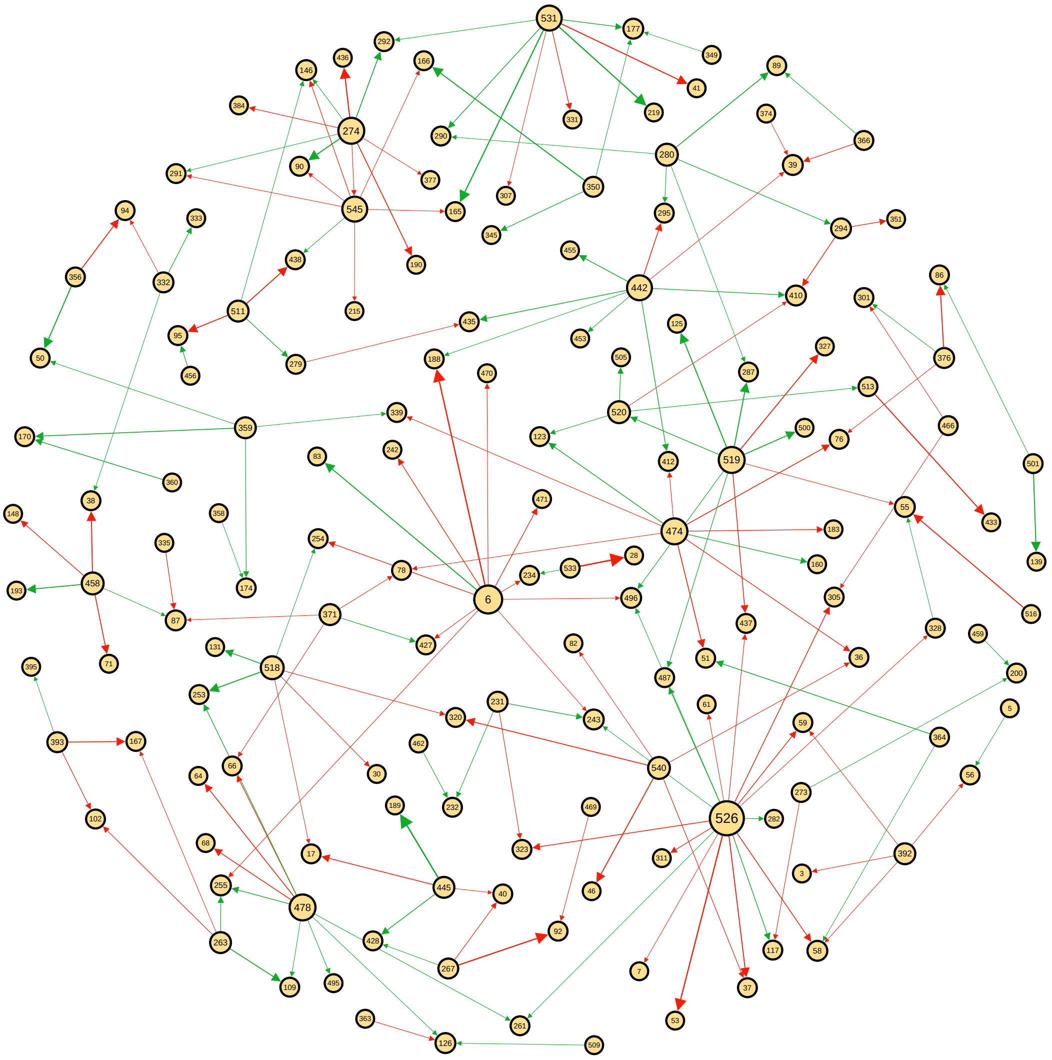

Supplementary Figure S4: Main regulatory clusters of Tzd2 state. Green links represent up-regulation interactions between nodes, while red links represent down-regulation interactions. The arrows indicate the direction of regulation, i.e., from regulator to regulated. The size of the cluster (circles) is proportional to the number of clusters that regulate. The size of the arrow is proportional to the fraction of the activity of regulated cluster explained by the influence of the regulator, i.e., proportional to $|w_{i,j}x_j|/|x_i|$.

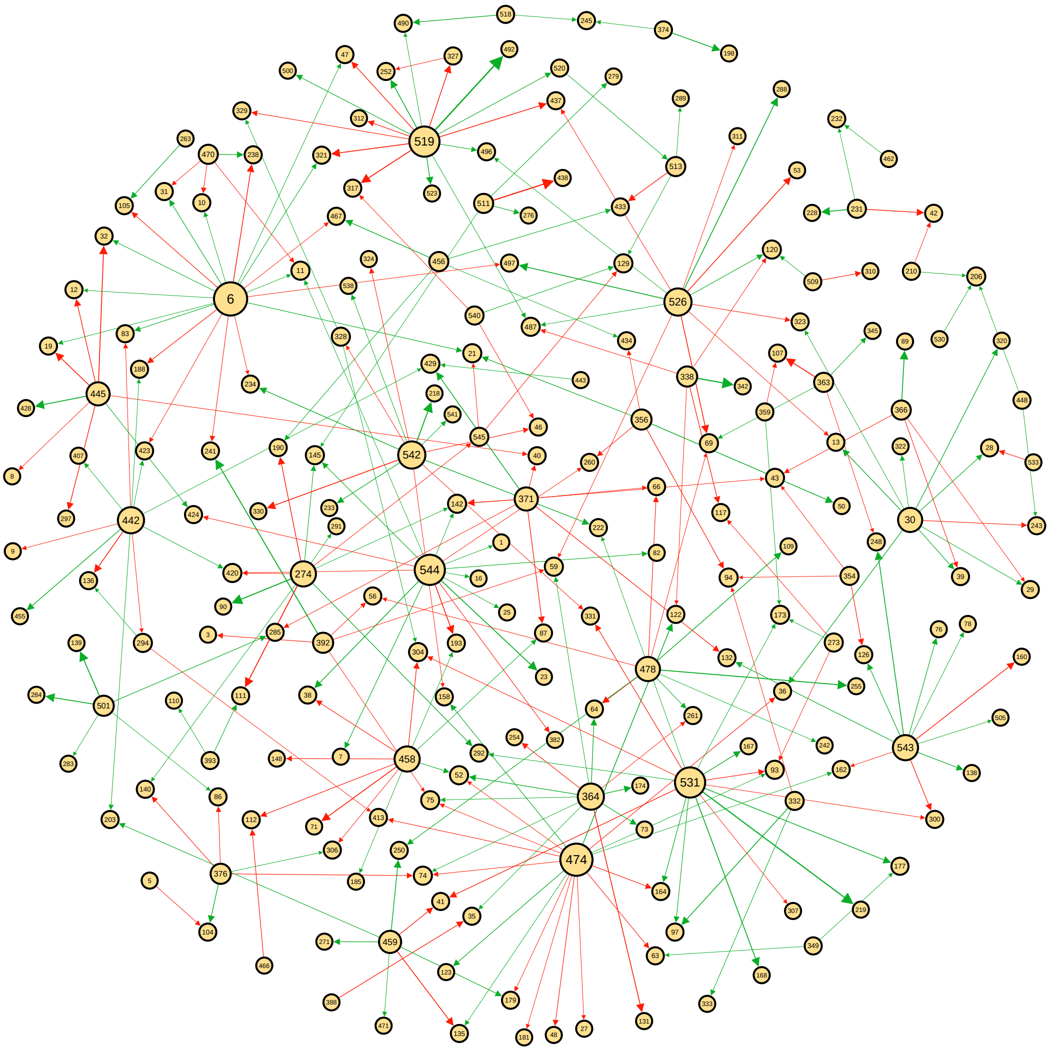

Supplementary Figure S5: Main regulatory clusters of Bzd21 state. Green links represent up-regulation interactions between nodes, while red links represent down-regulation interactions. The arrows indicate the direction of regulation, i.e., from regulator to regulated. The size of the cluster (circles) is proportional to the number of clusters that regulate. The size of the arrow is proportional to the fraction of the activity of regulated cluster explained by the influence of the regulator, i.e., proportional to $|w_{i,j}x_j|/|x_i|$.

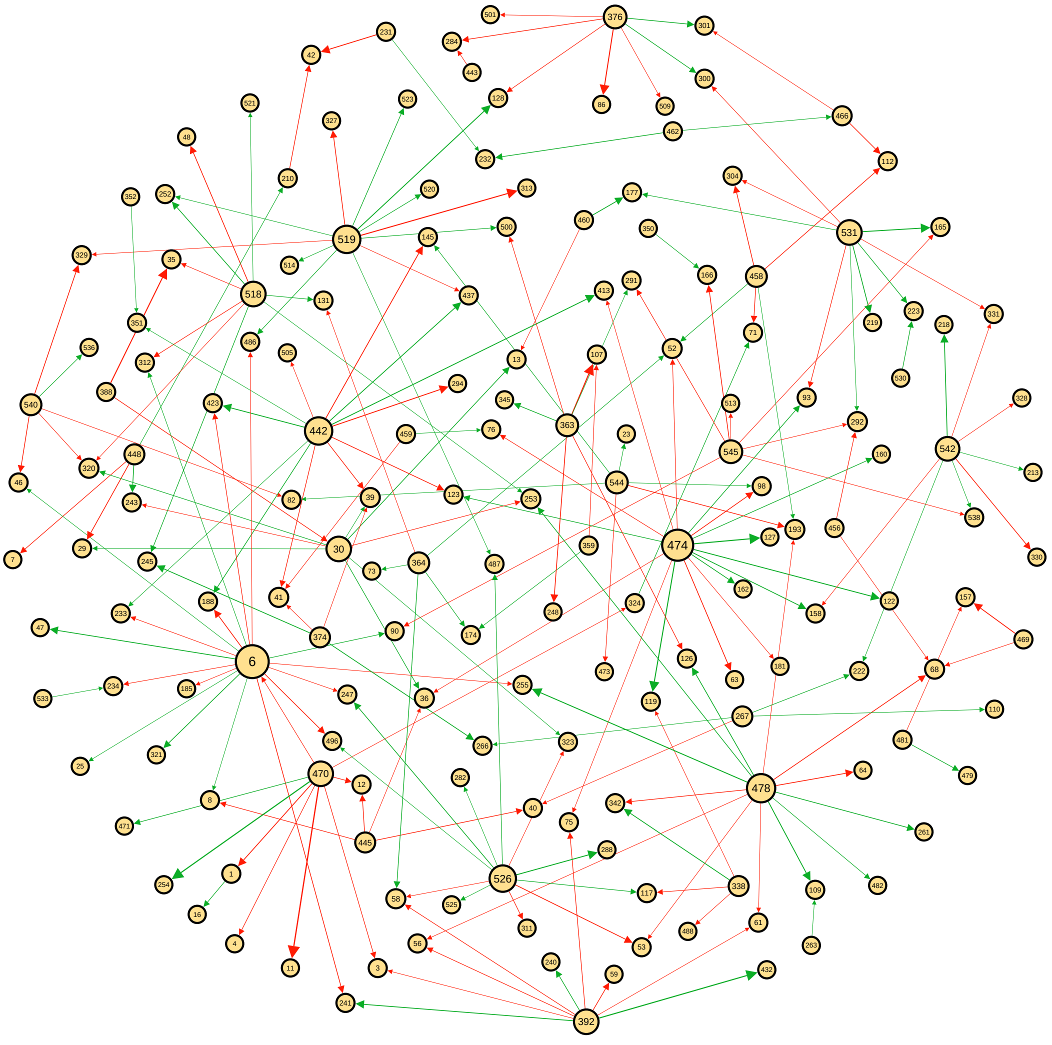

Supplementary Figure S6: Main regulatory clusters of Mc52 state.Green links represent up-regulation interactions between nodes, while red links represent down-regulation interactions. The arrows indicate the direction of regulation, i.e., from regulator to regulated. The size of the cluster (circles) is proportional to the number of clusters that regulate. The size of the arrow is proportional to the fraction of the activity of regulated cluster explained by the influence of the regulator, i.e., proportional to $|w_{i,j}x_j|/|x_i|$.

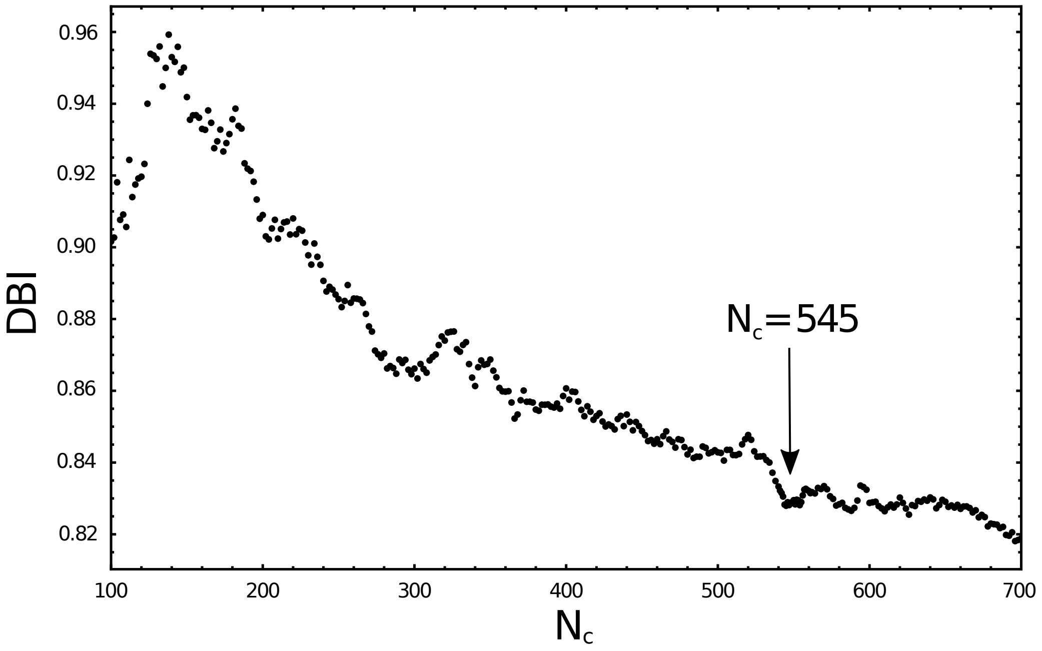

Supplementary Figure S7: Davies-Bouldin index as a function of the number of clusters N_c. The arrow in the local valley indicate the value of N_c used for further calculations.
